# Associations between psychological distress and untreated dental caries in conflict-affected Syrian adolescents: a cross-sectional study

**DOI:** 10.3389/froh.2026.1935552

**Published:** 2026-08-17

**Authors:** Rana AlKattan, Wail Habal, Muaaz Alkhouli, Zuhair Al-Nerabieah, Ramy Habal, Ahmad Al Masri, Mohammed Awawdeh

**Affiliations:** 1 Department of Preventive Dental Sciences, College of Dentistry, King Saud bin Abdulaziz University for Health Sciences, Riyadh, Saudi Arabia; 2 Ministry of National Guard Health Affairs, King Abdullah International Medical Research Center, Riyadh, Saudi Arabia; 3 Dental Services, King Abdulaziz Medical City, Ministry of National Guard Health Affairs, Riyadh, Saudi Arabia; 4 Department of Restorative and Cosmetic Dentistry, BPP University – Birmingham, Birmingham, United Kingdom; 5 School of Pharmacy & Pharmaceutical Sciences, Ulster University, Coleraine, United Kingdom; 6 Pediatric Dentistry, Arab International University, Daraa, Syria; 7 Faculty of Dentistry, Pediatric Dentistry, Damascus University, Damascus, Syria; 8 Department of Periodontology, BPP University – Birmingham, Birmingham, United Kingdom; 9 Department of Pediatric Dentistry, Department of Orthodontic Dentistry, Universitatsmedizin Greifswald, Greifswald, Germany; 10 College of Medicine & Dentistry, Ulster University, Birmingham, United Kingdom

**Keywords:** adolescents, dental caries, internally displaced, mental health, oral health, psychological distress, PTSD, Syria

## Abstract

**Introduction:**

Evidence regarding the oral health of internally displaced Syrian adolescents remains limited. This study evaluated oral health status and examined the association between psychological distress and untreated dental caries among adolescents living in conflict-affected areas of Syria.

**Methods:**

This cross-sectional study included 99 adolescents aged 12–17 years. Clinical oral examinations were performed according to World Health Organization (WHO) criteria. Psychological status was assessed using validated Arabic versions of the International Trauma Questionnaire (ITQ), Depression Anxiety Stress Scale-21 (DASS-21), and Epworth Sleepiness Scale (ESS). Associations between untreated dental caries, psychological status, and socio-demographic characteristics were evaluated using descriptive statistics and multivariable regression analyses.

**Results:**

The mean DMFT score was 8.42 ± 6.31, with untreated caries accounting for most of the disease burden (mean decayed teeth = 7.44 ± 6.58). Urgent dental treatment was required in 30.3% of participants. Poorer dentition status was significantly associated with rural displacement and lower parental educational level. Importantly, symptoms of post-traumatic stress disorder (PTSD), disturbances in self-organization (DSO), complex PTSD, depression, anxiety, stress, and sleep disturbances remained significantly associated with greater untreated dental decay after adjustment for residential location and parents’ education level. Greater psychological symptom severity was associated with progressively higher decayed teeth scores.

**Conclusions:**

Conflict-affected Syrian adolescents exhibited a high burden of untreated dental disease and substantial unmet treatment needs. Although socioeconomic disadvantage was associated with poorer oral health, psychological distress remained significantly associated with greater untreated dental decay after adjustment for residential location and parental education. These findings support the integration of oral healthcare and psychosocial services to reduce oral health inequalities among adolescents living in conflict-affected settings.

## Introduction

Military conflict is associated with profound physical, psychological, and social consequences, particularly among children and adolescents. Exposure to war-related events has been linked to a wide range of psychological health problems, including post-traumatic stress disorder (PTSD), depression, anxiety, sleep disturbances, fear, aggressive behaviour, and impaired academic performance (1–3). Adolescents are especially vulnerable to these effects because of the critical developmental changes that occur during this period.

Psychological well-being is increasingly recognized as an important determinant of oral health. Mental health disorders can influence oral hygiene practices, dietary behaviours, healthcare-seeking patterns, and overall quality of life, thereby increasing the risk of oral disease (4). Oral health conditions such as untreated dental caries, traumatic dental injuries, and chronic pain may also negatively affect psychological well-being, creating a bidirectional relationship between mental and oral health (5, 6). Previous research has demonstrated that children experiencing psychological distress often report poorer oral health outcomes and reduced oral health-related quality of life (7).

The Syrian conflict, which has persisted for more than a decade, has resulted in widespread displacement, disruption of healthcare services, and deterioration of living conditions. These circumstances have contributed to substantial psychological health challenges among Syrian children and adolescents (8, 9). At the same time, studies have reported a high prevalence of untreated dental caries, poor oral hygiene, and limited access to dental care among Syrian children affected by the conflict (10–13). Furthermore, Syrian children diagnosed with PTSD have been shown to exhibit significantly poorer dental health and greater caries experience compared with unaffected children (14, 15).

Several mechanisms may explain the relationship between psychological distress and oral health in conflict settings. Depression, anxiety, and PTSD may reduce motivation to maintain oral hygiene, increase self-neglect, and limit utilization of preventive and restorative dental services (16). Dental treatment may also trigger anxiety or fear among individuals exposed to traumatic experiences, particularly when dental procedures evoke memories associated with conflict-related events (17). In addition, psychotropic medications commonly prescribed for psychological health disorders may adversely affect oral health through side effects such as xerostomia (18).

Despite growing evidence regarding the health consequences of war among Syrian refugees, considerably less attention has been directed toward children and adolescents who remain within conflict-affected regions of Syria (19). To our knowledge, this is among the first studies to simultaneously evaluate oral health status and multiple dimensions of psychological distress among internally displaced Syrian adolescents living within a conflict-affected setting. Internally displaced populations may face unique challenges related to healthcare access, socioeconomic instability, and ongoing exposure to adversity. Consequently, important gaps remain in understanding the relationship between psychological well-being and oral health among adolescents living in these settings.

Therefore, the aims of this study were to evaluate the dentition status of permanent teeth among Syrian adolescents living in conflict-affected areas and to investigate the association between socio-demographic characteristics, psychological health, and untreated dental caries. The null hypothesis was that decayed teeth would not be associated with demographic characteristics or psychological health indicators.

## Materials and methods

### Study design

This cross-sectional study investigated the association between psychological status and oral health among Syrian adolescents living in conflict-affected settings. Data collection comprised two components: a) a questionnaire-based assessment of psychological health and socio-demographic characteristics, and b) a clinical oral examination to evaluate dental health status.

### Participants

Adolescents aged 12–17 years attending participating private dental clinics in Damascus, Syria, between September and December 2021 were eligible for inclusion. Private dental clinics were selected for logistical reasons, as these facilities were accessible to the investigators and permitted standardized clinical examinations and questionnaire administration during the study period. Participants were recruited using consecutive sampling, whereby all adolescents meeting the eligibility criteria during the study period were invited to participate. The study was conducted during a period in which Syria continued to experience the prolonged effects of armed conflict, economic deterioration, and disruption of healthcare services. Although the intensity of active conflict had decreased in Damascus compared with earlier years, internally displaced populations continued to experience substantial socioeconomic hardship, limited access to healthcare, and ongoing psychological stress. These contextual factors provide important background for interpreting the health status of the study population.

Ethical approval for the study was granted by the College of Medicine and Dentistry, Birmingham, United Kingdom (BP0206148/250621) prior to participant recruitment. Administrative approval to conduct the study was obtained from the participating private dental clinics in Damascus, Syria. Written informed consent was obtained from a parent or legal guardian prior to enrolment, and all procedures were conducted in accordance with the principles of the Declaration of Helsinki. Participants younger than 12 years or older than 17 years, those who were medically unfit for examination, those with incomplete forms, and those unable to communicate in Arabic or English were excluded.

### Tools of the study

Six diagnostic tools were used in this study, which ranged from questionnaires to clinical examinations or a combination of both, for the assessment of psychological and dental health of the participants (Table 1).

**Table 1:** Diagnostic tools used in the study.

Psychological health and socio-demographic assessment	
International Trauma Questionnaire (ITQ)	Cloitre et al. (41)
Depression Anxiety Stress Scale-21 (DASS-21)	Lovibond and Lovibond (42)
Epworth Sleepiness Scale (ESS)	Johns (43)
World Health Organization (WHO) Oral Health Questionnaire for Children Annex 8, 5th edition	WHO (26)

Clinical Dental Health Examination	
World Health Organization (WHO) Oral Health Assessment Annex 2, 5th edition	WHO (26)
Modified DDE index for enamel defects screening	Clarkson and O’mullane (44)

### Psychological and socio-demographic assessment

Psychological health was assessed using validated Arabic versions of three standardized instruments. The Arabic versions of the International Trauma Questionnaire (ITQ), Depression Anxiety Stress Scale-21 (DASS-21), and Epworth Sleepiness Scale (ESS) have demonstrated good internal consistency (Cronbach's *α* > 0.80) and construct validity in previous validation studies involving Arabic-speaking populations. The ITQ was used to identify symptoms of post-traumatic stress disorder (PTSD), disturbances in self-organization (DSO), and complex PTSD (CPTSD) according to the International Classification of Diseases, 11th Revision (ICD-11), which has previously demonstrated good validity and reliability in children and adolescents (20). PTSD, DSO, and CPTSD were classified as positive or negative according to the ICD-11 diagnostic algorithm implemented within the ITQ.

Symptoms of depression, anxiety, and stress were assessed using the Arabic version of the DASS-21, which has been validated in both Arabic-speaking populations and adolescents (21–23). Scores were categorized into normal, mild, moderate, severe, and extremely severe according to the recommended DASS-21 severity cut-offs. Sleep quality was evaluated using the Arabic version of the ESS. The ESS has been validated for use in children and adolescents, and the Arabic version has demonstrated satisfactory reliability and validity (24, 25). ESS scores were categorized as normal (0–10), borderline (11–12), or abnormal (≥13) according to the established scoring guidelines.

Socio-demographic information was collected using the World Health Organization (WHO) Oral Health Questionnaire for Children (5th Edition, Annex 8). Participants completed the questionnaires independently, with assistance provided by trained examiners when required. Additional details regarding the psychological and socio-demographic assessment have been reported previously by Habal et al. (19). The present study represents a secondary analysis of the same cohort of participants. The previous publication reported the prevalence and sociodemographic correlates of psychological distress, whereas the current analysis investigates its relationship with clinically assessed oral health outcomes.

### Clinical oral examination

Clinical oral examinations were performed by a trained examiner (W.H.) in accordance with WHO Oral Health Survey Methods (5th Edition, Annex 2) (26). Permanent dentition status was assessed and recorded for all visible permanent teeth. The teeth were examined in an orderly manner from the last tooth in the upper right quadrant to the last tooth in the lower right quadrant. If primary and permanent teeth were present in the same space, only the permanent tooth was recorded. If any part of the tooth was visible, the tooth was considered present.

Each tooth was classified according to WHO criteria as sound, carious, restored with caries, restored without caries, sealed, missing/unerupted, or otherwise affected by dental disease following the WHO assessment form. The recorded data were subsequently used to calculate the Decayed, Missing, and Filled Teeth (DMFT) index for each participant.

Additional oral health outcomes included traumatic dental injuries, dental erosion, fluorosis, and treatment urgency were also assessed using WHO diagnostic criteria. Developmental enamel defects were recorded using the Modified Developmental Defects of Enamel (DDE) Index. Following examination, participants were informed of their oral health status and referred for appropriate dental care when indicated.

### Statistical analysis

Statistical analysis was conducted to explore the association between the demographics of the population, their psychological health and dentition status. The dentition status was assessed based on the World Health Organization (WHO) Oral Health Assessment, Annex 2, using a categorical indicator for each tooth [Missing/unerupted; Sound; Fissure sealant; Filled, no caries; Caries (restorable); Filled w/caries; Fixed dental prosthesis; Hopeless/unrestorable caries; Missing due to caries/trauma]. The recorded data were subsequently used to calculate the Decayed, Missing, and Filled Teeth (DMFT) index. The number of decayed (D) teeth was used as the primary dental outcome for analyses examining associations with demographic and psychological variables.

Descriptive statistics (mean/SD or frequency/percentage) were used to summarize key study variables. Relationship between demographic variables (age, gender, location, parents’ educational level) and the decayed (D) component of the DMFT index was explored using correlation, independent-samples *t*-test and one-way ANOVA (with Tukey's *post-hoc* tests).

Primary study objective (relationship between psychological health and dentition status) was explored using linear regression models. Unadjusted analysis was performed first examining bivariate relationship between psychological health variables and decayed teeth. Age, sex, residential location, and parental educational level were initially evaluated for associations with the number of decayed teeth. Residential location and parental educational level were significantly associated with the outcome and were therefore included as covariates in the adjusted regression models; age and sex were not significantly associated and were not retained.

Several measures were implemented to reduce potential sources of bias. Consecutive sampling of all eligible participants during the recruitment period was used to minimize selection bias. Standardized WHO examination criteria and validated Arabic versions of the psychological assessment instruments were used to reduce measurement bias, and all clinical examinations were performed by trained examiners following a standardized protocol. Potential confounding by residential location and parental educational level was addressed using adjusted multivariable regression analyses. All tests were carried out with a significance level 0.05 and performed using SPSS Statistics for Windows, Version 31.0 (IBM Corp., Armonk, NY).

## Results

### Socio-demographic data

A total of 99 participants were included in the study who completed both the questionnaire and clinical examination. No eligible participants were excluded after enrolment because of incomplete data. Participants’ age ranged from 12 to 17 years, with a mean age of 13.97 (SD = 1.53) years. The gender and location of the participants were divided almost equally among males (*n* = 51, 51.5%) and females (*n* = 48, 48.5%), as well as among urban (*n* = 36, 36.3%), peri-urban (*n* = 26, 26.3%) and rural (*n* = 37, 37.4%) areas. Majority of parents had a college or university degree (*n* = 59, 59.6%), followed by high school degree (*n* = 19, 19.2%), secondary school or less (*n* = 14, 14.1%), and no adult in the household or education level unknown (*n* = 7, 7.1%).

### Clinical dental health findings

**Figure 1:**
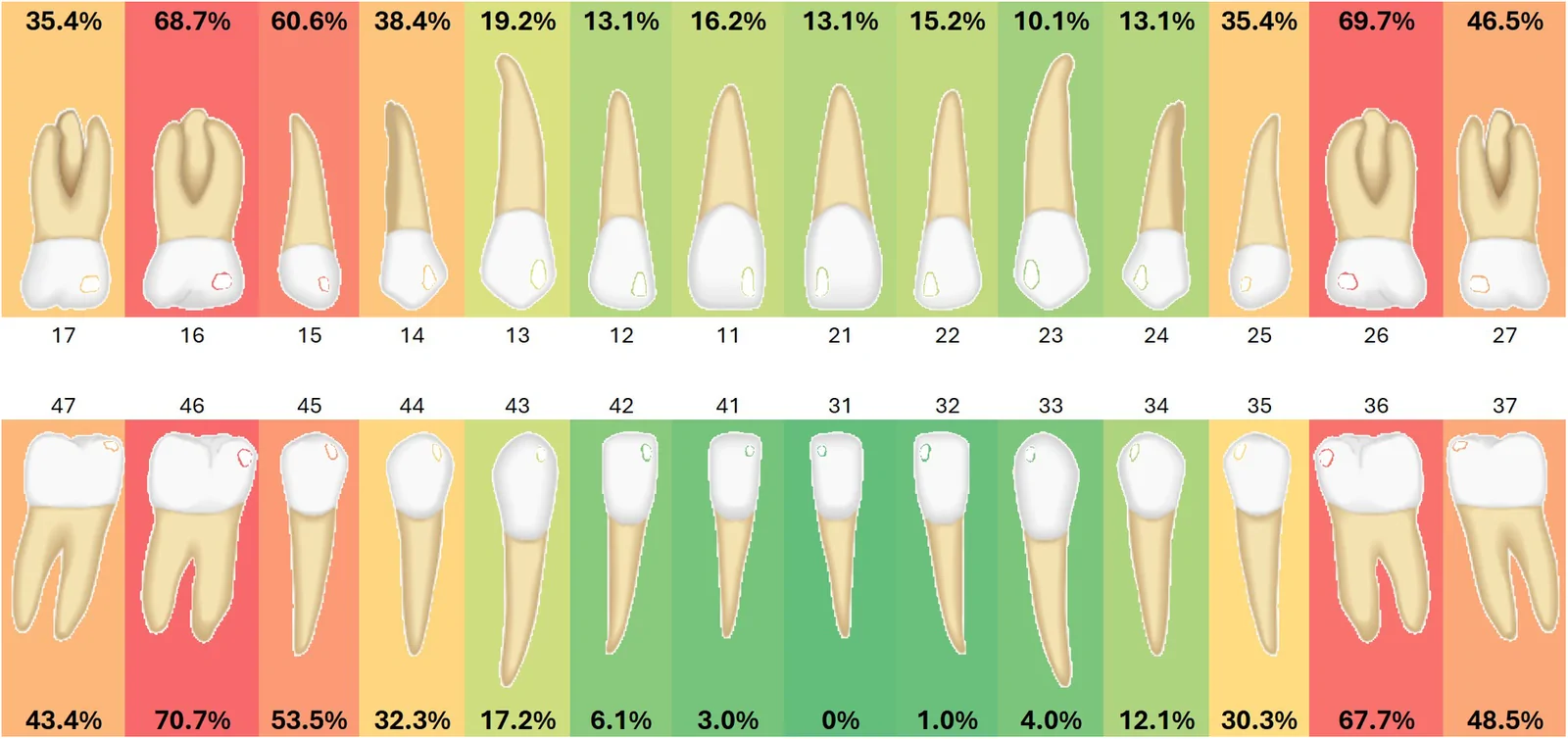
DMFT status using the international tooth numbering system (FDI).

The DMFT score of the participants ranged from 0 to 24 (with possible range 0–28) with a mean of 8.42 (SD = 6.31). Decayed, missing and filled teeth means were reported as 7.44 (SD = 6.58), 0.02 (SD = 0.14), and 0.96 (SD = 1.25), respectively. Only 18 participants (18.2%) reported no decayed teeth, and 11 (11.1%) reported a DMFT score of 0. Detailed data on DMFT status are shown in Figure 1.

The data in Table 2 summarizes the prevalence of traumatic dental injuries, fluorosis, erosion, intervention urgency, and DDE among the participants. The intervention urgency was based on the clinical examiner's judgment as the last question in the WHO assessment form following complete evaluation of the participants’ oral and dental health. Results indicated that 30 participants (30.3%) were in urgent need for treatment due to pain or infection in the oral region, with 3 participants (3.0%) requiring immediate referral for comprehensive evaluation due to a systemic condition. Fourteen (14.1%), 29 (29.3%), and 23 (23.2%) of the participants were judged as needing no treatment, preventive or routine treatment, or prompt treatment, respectively.

**Table 2:** The prevalence of traumatic dental injuries, fluorosis, erosion, intervention urgency, and DDE among participants.

Description	*N* (%)
Traumatic dental injuries	
No sign of injury	82 (82.8%)
Treated injury	3 (3.0%)
Fracture involving enamel only	9 (9.1%)
Fracture involving dentin	3 (3.0%)
Fracture leading to pulp involvement	0 (0%)
Missing tooth due to trauma	2 (2.0%)
Other damage	0 (0%)
Fluorosis^†^	
Normal	94 (94.9%)
Questionable	2 (2.0%)
Very mild	2 (2.0%)
Mild	0 (0%)
Moderate	1 (1.0%)
Severe	0 (0%)
Dental Erosion	
No sign of erosion	93 (93.9%)
Erosion involving enamel only	5 (5.1%)
Erosion reaching dentin	1 (1.0%)
Erosion leading to pulp involvement	0 (0%)
Intervention urgency	
No treatment needed	14 (14.1%)
Preventive or routine treatment needed	29 (29.3%)
Prompt treatment (including scaling) needed	23 (23.2%)
Immediate (urgent) treatment needed due to pain/infection	30 (30.3%)
Referred for comprehensive evaluation (systemic condition)	3 (3.0%)
Modified DDE index for enamel defects^1^	
Normal	74 (74.7%)
Demarcated opacity	12 (12.1%)
Diffuse opacity	2 (2.0%)
Hypoplasia	7 (7.1%)
Other defects	6 (6.1%)

Sum of percentages exceed 100% as patients can have multiple categories.

† Fluorosis: Normal, smooth, glossy enamel surface, Questionable, slight aberrations in the translucent normal enamel and a few white flecks with spots, Very mild, small, opaque, paper-white areas found on the tooth involving less than 25% of the labial surface, Mild, white patches in enamel involving more than 25% and less than 50% of the tooth surface, Moderate, wear of enamel surface is present with brown staining, Severe, severe hypoplasia and general form of the tooth is affected, with corroded appearance in general.

^1^Modified DDE index for enamel defects: Normal, no visible deviations from normal enamel translucency, smoothness, or thickness, Demarcated opacity, an alteration in the translucency of the enamel, varying in color (white, cream, yellow, or brown), with a clear, distinct boundary separating it from normal enamel, Diffuse opacity, an abnormality in enamel translucency characterized by a variable degree of cloudiness or linear/patchy patterns without a clear, sharp boundary with adjacent normal enamel, Hypoplasia, a quantitative defect representing partial or complete absence of enamel, appearing as pits, wide or narrow grooves, or larger missing areas, Other defects, Those not found in categories of demarcated opacity, diffuse opacity, or hypoplasia.

Enamel defects using the Modified DDE index demonstrated that the majority of participants had normal enamel (*n* = 74, 74.7%), while a demarcated opacity was recorded in 12 patients (12.1%), diffuse opacity in 2 patients (2.0%), hypoplasia in 7 patients (7.1%), and other enamel defects in 6 patients (6.1%).

### Association between demographic characteristics and dental health

Results in Table 3 demonstrated that two demographic factors (location and parents’ education) had a significant effect on the decayed teeth score (*p* < 0.001 and *p* < 0.001 respectively). Participants living in rural areas and those with parents of lower educational level had significantly more decayed teeth. However, the gender didn’t show any significant difference (*p* = 0.664). Also, age had no significant association with decayed score, *r*(99) = −0.01, *p* = 0.892. Adjusted models will thus only include the two significant covariates—location and parents’ education.

**Table 3:** Relationship between demographic data and decayed (D) teeth score.

Demographic factors	Decayed (D) component score	
	Mean (SD)	*P*-value
Gender		0.664
Male	7.73 (6.70)	
Female	7.15 (6.51)	
Location		<0.001
Urban	4.42 (4.20)^a^	
Peri-urban	6.62 (6.87)^a^	
Rural	10.97 (6.74)^b^	
Parents educational level		<0.001
Secondary school or less	13.36 (6.58)^b^	
High school completed	10.79 (6.65)^b^	
College/University completed	4.83 (5.31)^a^	
No adult in household/don’t know	8.57 (4.76)^a^^,^^b^	

a,b,c Different lowercase letters indicate significant differences in *post-hoc* testing.

### Association between psychological health and dental health

Considering location and parents’ educational level had significant association with decayed score and were significantly associated with psychological health (19), they were used as covariates to control for in adjusted regression analysis. The more severe the depression, anxiety, and stress, and the more abnormal the sleep, the greater the untreated dental caries. Moreover, participants with positive PTSD, DSO, and CPTSD symptoms according to the ITQ had significantly more untreated dental caries (Table 4). These findings remain significant in adjusted models (with location and parents’ education often losing significance in the adjusted models). This suggests psychological health being a significant factor with regards to dental health even when controlling for location and education of parents.

**Table 4:** Relationship between DASS, ESS, ITQ and dental health measured by decayed (D) teeth score.

Psychological health	*n*	Decayed (D) score	Unadjusted model		Adjusted^†^ model	
		Mean (SD)	*B* (SE), *p*	95% CI	*B* (SE), *p*	95% CI
Depression Diagnosis Scale			*P* < 0.001		*P* < 0.001	
Normal	25	1.40 (2.60)	reference	−1.85, 5.05	reference	−2.64, 4.53
Mild	9	3.00 (3.39)	1.60 (1.74), *p* = 0.359	2.33, 7.51	0.94 (1.80), *p* = 0.603	1.53, 6.83
Moderate	22	6.32 (3.90)	4.92 (1.31), *p* < 0.001	5.28, 11.20	4.18 (1.33), *p* = 0.002	3.54, 10.08
Severe	14	9.64 (5.88)	8.24 (1.49), *p* < 0.001	10.01, 14.85	6.81 (1.65), *p* < 0.001	7.53, 13.34
Extremely severe	29	13.83 (5.52)	12.43 (1.22), *p* < 0.001		10.43 (1.46), *p* < 0.001	
Anxiety Diagnosis Scale			*P* < 0.001		*P* < 0.001	
Normal	23	1.65 (2.85)	reference	−3.71, 3.61	reference	−3.94, 2.91
Mild	10	1.60 (1.90)	−0.05 (1.84), *p* = 0.977	−3.14, 7.33	−0.52 (1.72), *p* = 0.765	−4.05, 5.79
Moderate	4	3.75 (4.79)	2.10 (2.64), *p* = 0.428	0.46, 8.81	0.87 (2.47), *p* = 0.726	−1.62, 6.70
Severe	7	6.29 (3.50)	4.63 (2.10), *p* = 0.030	7.29, 12.09	2.54 (2.09), *p* = 0.228	5.36, 10.53
Extremely severe	55	11.35 (5.89)	9.69 (1.21), *p* < 0.001		7.95 (1.30), *p* < 0.001	
Stress Diagnosis Scale			*P* < 0.001		*P* < 0.001	
Normal	41	2.27 (3.23)	reference	1.38, 8.41	reference	2.40, 9.71
Mild	6	7.17 (3.97)	4.90 (1.77), *p* = 0.007	3.21, 7.34	6.06 (1.84), *p* = 0.001	2.80, 7.05
Moderate	24	7.54 (3.92)	5.27 (1.04), *p* < 0.001	9.97, 14.82	4.93 (1.07), *p* < 0.001	8.92, 14.20
Severe	15	14.67 (6.09)	12.40 (1.22), *p* < 0.001	10.56, 15.68	11.56 (1.33), *p* < 0.001	8.33, 14.18
Extremely severe	13	15.38 (3.80)	13.12 (1.29), *p* < 0.001		11.25 (1.47), *p* < 0.001	
Epworth Sleepiness Scale			*P* < 0.001		*P* < 0.001	
Normal	57	4.07 (4.73)	reference	0.86, 6.72	reference	−1.40, 4.83
Borderline	14	7.86 (5.23)	3.79 (1.48), *p* = 0.012	7.77, 12.30	1.72 (1.57), *p* = 0.277	5.15, 10.62
Abnormal	28	14.11 (5.24)	10.04 (1.14), *p* < 0.001		7.89 (1.38), *p* < 0.001	
Diagnoses based on ITQ						
PTSD			*P* < 0.001		*P* < 0.001	
Negative	47	2.77 (3.47)	reference	6.97, 10.85	reference	5.09, 9.21
Positive	52	11.67 (5.82)	8.91 (0.98), *p* < 0.001		7.15 (1.04), *p* < 0.001	
DSO			*P* < 0.001		*P* < 0.001	
Negative	48	2.75 (3.34)	reference	7.22, 11.01	reference	5.52, 9.44
Positive	51	11.86 (5.77)	9.11 (0.96), *p* < 0.001		7.48 (0.99), *p* < 0.001	
CPTSD			*P* < 0.001		*P* < 0.001	
Negative	52	3.08 (3.58)	reference	7.32, 11.08	reference	5.47, 9.53
Positive	47	12.28 (5.71)	9.20 (0.95), *p* < 0.001		7.50 (1.02), *p* < 0.001	

SD, standard deviation; *B*, regression coefficient; SE, standard error; CI, confidence interval for regression coefficients.

Location *p*-values in 7 adjusted models: 0.595, 0.137, 0.475, 0.373, 0.081, 0.048, 0.143.

Parents’ education *p*-values in 7 adjusted models: 0.215, 0.023, 0.039, 0.072, 0.078, 0.061, 0.058.

† Adjusted for location and parents’ education level.

The results of correlation analysis demonstrated that untreated dental caries showed a strong positive relationship with depression, anxiety, stress, and sleep quality (*p* < 0.001). Furthermore, unadjusted and adjusted regression models show only psychological health variables were significantly associated with a greater number of decayed teeth, while location and parents’ education were no longer significant (Table 5).

**Table 5:** Correlation between DASS, ESS scores and dental health measured by decayed (D) teeth score.

Psychological health	Bivariate correlation with decayed (D) score	Unadjusted model		Adjusted^†^ model	
	*r*, *p*	*B* (SE), *p*	95% CI	*B* (SE), *p*	95% CI
Depression scale	*r* = 0.734, *p* < 0.001	0.78 (0.07), *p* < 0.001	0.64, 0.93	0.67 (0.09), *p* < 0.001	0.49, 0.84
Anxiety scale	*r* = 0.741, *p* < 0.001	0.83 (0.08), *p* < 0.001	0.68, 0.98	0.71 (0.09), *p* < 0.001	0.53, 0.89
Stress scale	*r* = 0.783, *p* < 0.001	0.90 (0.07), *p* < 0.001	0.76, 1.04	0.80 (0.09), *p* < 0.001	0.62, 0.97
DASS 21 total score	*r* = 0.768, *p* < 0.001	0.29 (0.02), *p* < 0.001	0.24, 0.34	0.26 (0.03), *p* < 0.001	0.20, 0.32
Epworth sleepiness scale	*r* = 0.729, *p* < 0.001	0.82 (0.08), *p* < 0.001	0.66, 0.97	0.70 (0.10), *p* < 0.001	0.50, 0.91

*r*, correlation coefficient; *B*, regression coefficient; SE, standard error; CI, confidence interval for regression coefficients.

Location *p*-values in 5 adjusted models: 0.833, 0.509, 0.700, 0.758, 0.717.

Parents’ education *p*-values in 5 adjusted models: 0.063, 0.124, 0.150, 0.131, 0.509.

† Adjusted for location and parents’ education level.

## Discussion

This study identified a substantial burden of untreated dental disease among conflict-affected Syrian adolescents and demonstrated that psychological distress was significantly associated with poorer oral health. Symptoms of PTSD, DSO, complex PTSD, depression, anxiety, stress, and sleep disturbances were all significantly associated with worse dental health. Importantly, these associations remained significant after adjustment for residential location and parental educational level, suggesting that psychological distress may contribute to poorer oral health beyond the measured socioeconomic determinants included in the present analysis. Consequently, the null hypothesis was rejected. Together, these findings highlight both the considerable unmet oral healthcare needs and the important role of psychological distress in shaping oral health among conflict-affected adolescents.

The present study demonstrated a considerable burden of untreated dental disease among conflict-affected Syrian adolescents. The mean DMFT score was 8.42, with untreated caries accounting for most of the disease burden, whereas only a small proportion of affected teeth had received restorative treatment. According to the results in Figure 1, permanent first molars were the teeth most likely affected by caries, reflecting their early eruption, prolonged exposure to the oral environment, and greater susceptibility to plaque accumulation during adolescence, while mandibular anterior teeth were the least. These findings should be interpreted within the context of the prolonged Syrian conflict. Prolonged disruption to dental services, socioeconomic hardship, and reduced access to preventive oral healthcare are potential contributors to poorer oral health during the Syrian conflict, however, these factors were not directly measured in the present study.

The observed results are consistent with previous studies involving Syrian refugee and conflict-affected populations, which have similarly reported high levels of untreated caries, with less than 10% of teeth restored in both primary and permanent dentitions, and limited restorative care (27, 28). The limited availability of community water fluoridation in Syria may also have contributed to the high burden of untreated caries (29), although fluorosis remained uncommon in the present cohort. Comparable studies conducted in Damascus before and after the onset of the Syrian conflict have also documented a marked increase in untreated dental caries following the deterioration of healthcare infrastructure (29, 30). Collectively, these findings place the substantial oral disease burden observed in the present cohort within the broader context of previously reported oral health challenges among Syrian children and adolescents affected by prolonged conflict. As participants were recruited from private dental clinics, the reported burden of untreated dental disease may overestimate that of the wider internally displaced adolescent population and should therefore be interpreted with appropriate caution.

The high prevalence of untreated dental disease translated into considerable treatment needs within this population. Nearly one-third of participants required urgent dental care because of pain or infection, indicating that oral disease had progressed beyond the preventive stage for many adolescents, and highlighting substantial unmet oral healthcare needs. Untreated dental conditions during adolescence may adversely affect nutrition, school attendance, daily functioning, and overall quality of life (19). These findings are consistent with previous studies among Syrian refugee populations, where dental pain and untreated oral disease have been identified as major contributors to feelings of helplessness for both children and their caregivers (31). They also served as major triggers for distress and frustration (32). The findings emphasize the need to strengthen preventive and restorative dental services as part of humanitarian health responses for conflict-affected adolescents.

Traumatic dental injuries were observed in 17.1% of participants, exceeding the prevalence reported among children living in non-conflict settings within the region (33–35). Although most traumatic dental injuries in children result from accidental falls, the higher prevalence observed in the present study should be interpreted cautiously. It has been suggested that increased exposure to environmental hazards and psychological distress associated with prolonged conflict could increase the risk of traumatic dental injuries (36, 37), however, these factors were not directly investigated in the present study. Developmental enamel defects were also observed and may increase susceptibility to fracture because of compromised enamel structure (38). Although these findings were secondary outcomes, they further illustrate the range of oral health conditions observed in this conflict-affected adolescent cohort beyond dental caries alone.

The principal contribution of this study lies in demonstrating that psychological distress was independently associated with poorer oral health among conflict-affected Syrian adolescents after adjustment for residential location and parental educational level. Participants with symptoms of PTSD, DSO, complex PTSD, depression, anxiety, stress, and sleep disturbances consistently exhibited significantly higher decayed teeth scores than those without these symptoms. These findings are consistent with previous reports demonstrating poorer oral health among Syrian children with PTSD and among refugee populations exposed to conflict (10, 11). Hamid and Dashash reported significantly poorer dental health and greater caries experience among Syrian children with PTSD than among children without PTSD (10). Similarly, Solyman and Schmidt reported a high prevalence of untreated dental caries among recently arrived Syrian and Iraqi refugees in Germany, suggesting that poor oral health is a persistent challenge among conflict-affected populations irrespective of the host setting (11). However, the present study extends existing evidence by simultaneously evaluating multiple dimensions of psychological distress and demonstrating that their associations with oral health persist after controlling for important demographic confounders. Furthermore, the present investigation focused on internally displaced adolescents who remained within Syria, demonstrating that substantial oral health inequalities persist even among conflict-affected populations who have not crossed international borders. Collectively, these findings suggest that psychological distress may represent an important factor associated with oral health inequalities among conflict-affected adolescents rather than merely reflecting underlying socioeconomic disadvantage (14, 19).

Participants with PTSD demonstrated an adjusted increase in the number of decayed teeth of approximately 7.15 compared with those without PTSD, highlighting the substantial clinical magnitude of this association. Several mechanisms may explain the observed relationship between psychological and oral health. Adolescents experiencing depression, anxiety, or post-traumatic stress may have reduced motivation to maintain oral hygiene, poorer dietary habits, lower utilization of preventive dental services, and increased avoidance of dental treatment because of fear or traumatic memories associated with healthcare environments (39). Chronic psychological stress may also influence immune and inflammatory responses, potentially exacerbating oral disease (40). In addition, psychotropic medications prescribed for psychological disorders may contribute to xerostomia, thereby increasing susceptibility to dental caries (18). Although the cross-sectional design precludes causal inference, the persistence of these associations after adjustment for socioeconomic factors suggests that psychological distress may play an important role in oral health inequalities in conflict-affected populations. Nonetheless, the findings should be interpreted within the context of the prolonged Syrian conflict. Although conflict-related factors such as displacement, healthcare disruption, and socioeconomic hardship may plausibly influence both psychological distress and oral health, these exposures were not directly measured in the present study and therefore should be regarded as contextual explanations rather than findings derived from our data.

This study has several limitations that should be considered when interpreting the findings. First, the cross-sectional design precludes establishing causal relationships between psychological and oral health, and the observed associations should therefore be interpreted with caution. Second, participants were recruited from private dental clinics in Damascus using consecutive sampling, which reflects logistical considerations and investigator access during the study period. As these adolescents were seeking dental care, they may have had greater treatment needs than adolescents in the general population, potentially introducing selection bias and limiting the generalizability of the findings. Furthermore, access to private dental clinics may vary according to socioeconomic circumstances and the study population may therefore not fully represent all internally displaced adolescents or those who rely on other healthcare settings. Third, the relatively modest sample size and recruitment from a single geographic region may further restrict the external validity of the results. Finally, psychological status was assessed using validated self-reported questionnaires rather than clinical psychiatric diagnoses, which may be subject to reporting bias. Nevertheless, the use of standardized and validated assessment instruments and adjustment for important demographic confounders strengthens the reliability of the observed associations. Future multicentre, population-based longitudinal studies are warranted to confirm these findings and to clarify the temporal and potentially causal relationship between psychological distress and oral health among conflict-affected adolescents.

In conclusion, conflict-affected Syrian adolescents attending private dental clinics exhibited a substantial burden of untreated dental disease and unmet treatment needs. Although these findings may not fully represent the wider internally displaced adolescent population, they highlight important oral health challenges among adolescents seeking dental care in conflict-affected settings. Greater untreated dental decay was associated with socioeconomic disadvantage, particularly rural displacement and lower parental educational level. Importantly, psychological distress was significantly associated with greater untreated dental decay after adjustment for these socioeconomic factors. Given the cross-sectional design and private clinic-based sample, these findings should be regarded as hypothesis-generating and warrant confirmation in larger, population-based longitudinal studies. Nevertheless, they highlight the potential value of considering psychological well-being alongside oral healthcare needs among adolescents living in conflict-affected settings.

## Data Availability

The original contributions presented in the study are included in the article/Supplementary Material, further inquiries can be directed to the corresponding authors.

## References

[1] Murthy RS Lakshminarayana R. Mental health consequences of war: a brief review of research findings. World Psychiatry. (2006) 5(1):25–30. PMID: 16757987. 16757987 PMC1472271

[2] Michelle Liu MD. War and children. Am J Psychiatry Resid J. (2017) 12(7):3–5. 10.1176/appi.ajp-rj.2017.120702

[3] Chrisman AK Dougherty JG. Mass trauma: disasters, terrorism, and war. Child Adolesc Psychiatr Clin N Am. (2014) 23:257–79. 10.1016/j.chc.2013.12.004 24656579

[4] Barasuol JC Santos PS Moccelini BS Magno MB Bolan M Martins-Júnior PA. Association between dental pain and oral health-related quality of life in children and adolescents: a systematic review and meta-analysis. Community Dent Oral Epidemiol. (2020) 48(4):257–63. 10.1111/cdoe.12535 32383273

[5] Feldens CA Ardenghi TM Dos Santos Dullius AI Vargas-Ferreira F Hernandez PAG Kramer PF. Clarifying the impact of untreated and treated dental caries on oral health-related quality of life among adolescents. Caries Res. (2016) 50(4):414–21. 10.1159/000447095 27458722

[6] Baldiotti ALP Amaral-Freitas G Barbosa MCF Moreira PR Machado RA Coletta RD. Associations between anxiety, depression, chronic pain and oral health-related quality of life, happiness, and polymorphisms in Adolescents’ genes. Int J Environ Res Public Health. (2023) 20(4):3321. 10.3390/ijerph20043321 36834016 PMC9967116

[7] Moghaddam LF Vettore MV Bayani A Bayat A-H Ahounbar E Hemmat M. The association of oral health status, demographic characteristics and socioeconomic determinants with oral health-related quality of life among children: a systematic review and meta-analysis. BMC Pediatr. (2020) 20(1):489. 10.1186/s12887-020-02371-8 33092562 PMC7579886

[8] Perkins JD Ajeeb M Fadel L Saleh G. Mental health in Syrian children with a focus on post-traumatic stress: a cross-sectional study from Syrian schools. Soc Psychiatry Psychiatr Epidemiol. (2018) 53(11):1231–9. 10.1007/s00127-018-1573-3 30083987 PMC6208941

[9] Kampalath V Tarakji A Hamze M Loutfi R Cohn K Abbara A. The impacts of the Syrian conflict on child and adolescent health: a scoping review. J Public Health. (2023) 45(3):621–30. 10.1093/pubmed/fdac132 36418238

[10] Hamid SH Dashash MAD. The impact of post-traumatic stress disorder on dental and gingival status of children during Syrian crisis: a preliminary study. J Investig Clin Dent. (2019) 10(1):e12372. 10.1111/jicd.12372 30460768

[11] Solyman M Schmidt-Westhausen AM. Oral health status among newly arrived refugees in Germany: a cross-sectional study. BMC Oral Health. (2018) 18(1):132. 10.1186/s12903-018-0600-9 30075766 PMC6091105

[12] Joury E. Syria profile of the epidemiology and management of early childhood caries before and during the time of crisis. Front Public Health. (2019) 7:271. 10.3389/fpubh.2019.00271 31612125 PMC6768970

[13] Riatto SG Montero J Pérez DR Castaño-Séiquer A Dib A. Oral health Status of Syrian children in the refugee center of melilla, Spain. Int J Dent. (2018) 2018:2637508. 10.1155/2018/2637508 29743889 PMC5878884

[14] Alhaffar MHDBA Janos S. Public health consequences after ten years of the Syrian crisis: a literature review. Global Health. (2021) 17(1):111. 10.1186/s12992-021-00762-9 34538248 PMC8449996

[15] Kakaje A Al Zohbi R Hosam Aldeen O Makki L Alyousbashi A Alhaffar MBA. Mental disorder and PTSD in Syria during wartime: a nationwide crisis. BMC Psychiatry. (2021) 21(1):2. 10.1186/s12888-020-03002-3 33388026 PMC7778805

[16] Raja S Hoersch M Rajagopalan CF Chang P. Treating patients with traumatic life experiences: providing trauma-informed care. J Am Dent Assoc. (2014) 145(3):238–45. 10.14219/jada.2013.30 24583888

[17] Cormac I Jenkins P. Understanding the importance of oral health in psychiatric patients. Adv Psychiatr Treat. (1999) 5(1):53–60. 10.1192/apt.5.1.53

[18] Lalloo R Kisely S Amarasinghe H Perera R Johnson N. Oral health of patients on psychotropic medications: a study of outpatients in Queensland. Australas Psychiatry. (2013) 21(4):338–42. 10.1177/1039856213486308 23671224

[19] Habal W Alkattan R Hajeer MY Alkhouli M Al-Nerabieah Z Habal T. Impact of Syrian conflict on the oral health of adolescents: a cross-sectional study. Cureus. (2024) 16(2):e54613. 10.7759/cureus.54613 38389568 PMC10883722

[20] Haselgruber A Sölva K Lueger-Schuster B. Validation of ICD-11 PTSD and complex PTSD in foster children using the International Trauma Questionnaire. Acta Psychiatr Scand. (2020) 141(1):60–73. 10.1111/acps.13100 31536646 PMC6973040

[21] Ali AM Green J. Factor structure of the depression anxiety stress scale-21 (DASS-21): unidimensionality of the Arabic version among Egyptian drug users. Subst Abuse Treat Prev Policy. (2019) 14(1):40. 10.1186/s13011-019-0226-1 31533766 PMC6751677

[22] Evans L Haeberlein K Chang A Handal P. Convergent validity and preliminary cut-off scores for the anxiety and depression subscales of the DASS-21 in US adolescents. Child Psychiatry Hum Dev. (2021) 52(4):579–85. 10.1007/s10578-020-01050-0 32816139

[23] Evans L Haeberlein K Chang A Handal P. An evaluation of the convergent validity of and preliminary cutoff scores for the DASS-21 total score as a measure of distress in adolescents. Curr Psychol. (2022) 41(7):4283–90. 10.1007/s12144-020-00937-4

[24] Ahmed AE Fatani A Al-Harbi A Al-Shimemeri A Ali YZ Baharoon S. Validation of the Arabic version of the Epworth sleepiness scale. J Epidemiol Glob Health. (2014) 4(4):297–302. 10.1016/j.jegh.2014.04.004 25455647 PMC7320333

[25] Janssen KC Phillipson S O'Connor J Johns MW. Validation of the epworth sleepiness scale for children and adolescents using rasch analysis. Sleep Med. (2017) 33:30–5. 10.1016/j.sleep.2017.01.014 28449902

[26] Petersen PE Baez RJ, World Health Organization. Oral Health Surveys: Basic Methods. 5th ed. Geneva: World Health Organization (2013).

[27] Salim NA Shaini FJ Sartawi S Al-Shboul B. Oral health Status and dental treatment needs in Syrian refugee children in zaatari camp. J Refug Stud. (2021) 34(2):2492–507. 10.1093/jrs/feaa133

[28] Makan R Gara M Awwad MA Hassona Y. The oral health status of Syrian refugee children in Jordan: an exploratory study. Spec Care Dentist. (2019) 39(3):306–9. 10.1111/scd.12377 30985010

[29] Beiruti N, Taifour D, van Palenstein Helderman WH, Frencken JE. A review of the oral health status in Syria. *Int Dent J.* (2001) 51(1):7–10. 10.1002/j.1875-595x.2001.tb00810.x 11326452

[30] Ballouk MA-H Dashash M. Caries prevalence and dental health of 8–12 year-old children in Damascus city in Syria during the Syrian crisis; a cross-sectional epidemiological oral health survey. BMC Oral Health. (2019) 19:1–6. 10.1186/s12903-019-0713-9 30646889 PMC6332908

[31] Pani SC Al-Sibai SA Rao AS Kazimoglu SN Mosadomi HA. Parental perception of oral health-related quality of life of Syrian refugee children. J Int Soc Prev Community Dent. (2017) 7(4):191–6. 10.4103/jispcd.JISPCD_212_17 28852635 PMC5558253

[32] Abu-Awwad M Al-Omoush S Shqaidef A Hilal N Hassona Y. Oral health-related quality of life among Syrian refugees in Jordan: a cross-sectional study. Int Dent J. (2020) 70(1):45–52. 10.1111/idj.12521 31489618 PMC9379194

[33] Rajab LD Baqain ZH Abu Ghazaleh SB Sonbol HN Hamdan MA. Traumatic dental injuries among 12-year-old schoolchildren in Jordan: prevalence, risk factors and treatment need. Oral Health Prev Dent. (2013) 11(2):105–12. 10.3290/j.ohpd.a29362 23534038

[34] Yassen GH Chin JR Al-Rawi BAO Mohammedsharif AG Alsoufy SS Hassan LA-R. Traumatic injuries of permanent teeth among 6-to 12-year-old Iraqi children: a 4-year retrospective study. J Dent Child. (2013) 80(1):3–8. PMID: 23595237. 23595237

[35] Fisher-Owens SA Gansky SA Platt LJ Weintraub JA Soobader M-J Bramlett MD. Influences on children’s oral health: a conceptual model. Pediatrics. (2007) 120(3):e510–20. 10.1542/peds.2006-3084 17766495

[36] Glendor U. Aetiology and risk factors related to traumatic dental injuries—a review of the literature. Dent Traumatol. (2009) 25(1):19–31. 10.1111/j.1600-9657.2008.00694.x 19208007

[37] Alsayed Tolibah Y Bshara N Makieh RE Alhaji M Al-Shiekh MN AlMonakel MHDB. Epidemiology meets advocacy: understanding pediatric dental trauma and delayed care in post-conflict Syria. Int J Environ Res Public Health. (2025) 22(12):1864. 10.3390/ijerph22121864 41464497 PMC12733264

[38] Seow W. Developmental defects of enamel and dentine: challenges for basic science research and clinical management. Aust Dent J. (2014) 59(Suppl 1):143–54. 10.1111/adj.12104 24164394

[39] Kisely S Baghaie H Lalloo R Siskind D Johnson NW. A systematic review and meta-analysis of the association between poor oral health and severe mental illness. Psychosom Med. (2015) 77(1):83–92. 10.1097/psy.0000000000000135 25526527

[40] Macrì M D’Albis G D’Albis V Antonacci A Abbinante A Stefanelli R. Periodontal health and its relationship with psychological stress: a cross-sectional study. J Clin Med. (2024) 13(10):2942. 10.3390/jcm13102942 38792482 PMC11122378

[41] Cloitre M Shevlin M Brewin CR Bisson JI Roberts NP Maercker A. The international trauma questionnaire: development of a self-report measure of ICD-11 PTSD and complex PTSD. Acta Psychiatr Scand. (2018) 138(6):536–46. 10.1111/acps.12956 30178492

[42] Lovibond PF Lovibond SH. The structure of negative emotional states: comparison of the depression anxiety stress scales (DASS) with the beck depression and anxiety inventories. Behav Res Ther. (1995) 33(3):335–43. 10.1016/0005-7967(94)00075-u 7726811

[43] Johns MW. A new method for measuring daytime sleepiness: the epworth sleepiness scale. Sleep. (1991) 14(6):540–5. 10.1093/sleep/14.6.540 1798888

[44] Clarkson J O'Mullane D. A modified DDE Index for use in epidemiological studies of enamel defects. J Dent Res. (1989) 68(3):445–50. 10.1177/00220345890680030201 2921385

